# The Cell-Cycle State of Stem Cells Determines Cell Fate Propensity

**DOI:** 10.1016/j.cell.2013.08.031

**Published:** 2013-09-26

**Authors:** Siim Pauklin, Ludovic Vallier

**Affiliations:** 1 Wellcome Trust-Medical Research Council Cambridge Stem Cell Institute, Anne McLaren Laboratory for Regenerative Medicine and Department of Surgery, University of Cambridge, Cambridge CB2 0SZ, UK; 2 Wellcome Trust Sanger Institute, Hinxton, Cambridge CB10 1SA, UK

## Abstract

Self-renewal and differentiation of stem cells are fundamentally associated with cell-cycle progression to enable tissue specification, organ homeostasis, and potentially tumorigenesis. However, technical challenges have impaired the study of the molecular interactions coordinating cell fate choice and cell-cycle progression. Here, we bypass these limitations by using the FUCCI reporter system in human pluripotent stem cells and show that their capacity of differentiation varies during the progression of their cell cycle. These mechanisms are governed by the cell-cycle regulators cyclin D1–3 that control differentiation signals such as the TGF-β-Smad2/3 pathway. Conversely, cell-cycle manipulation using a small molecule directs differentiation of hPSCs and provides an approach to generate cell types with a clinical interest. Our results demonstrate that cell fate decisions are tightly associated with the cell-cycle machinery and reveal insights in the mechanisms synchronizing differentiation and proliferation in developing tissues.

## Highlights

• Cell fate decisions are cell-cycle dependent
• Control of endoderm versus neuroectoderm differentiation by cyclin D-CDK4/6
• Cyclin Ds control TGF-β-Smad2/3 transcriptional activity
• Differentiation of hPSCs can be directed by manipulation of the cell cycle

## Graphical Abstract

**Figure:**
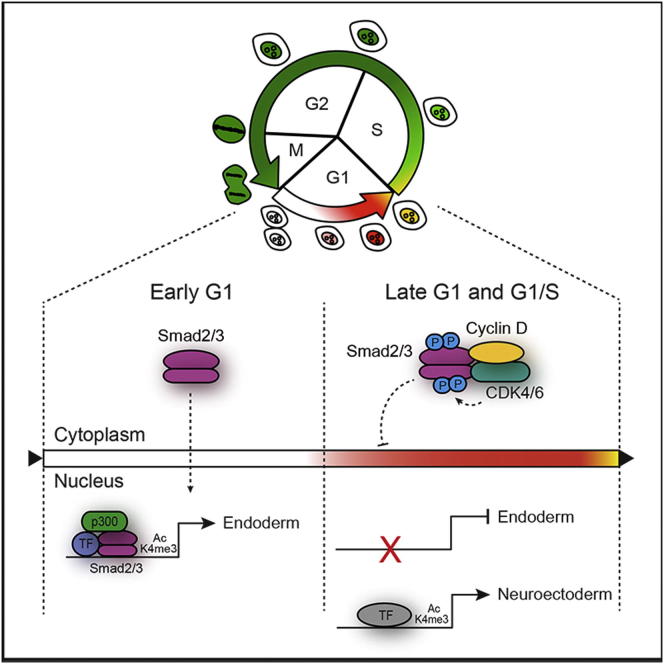

## Teaser

Cell fate decisions are influenced by the cell-cycle state and cyclin D activity of a stem cell as it embarks on differentiation. Cyclin D acts via TGF-β-Smad signaling to alter the propensity for endoderm versus neuroectoderm fate.

## Introduction

Tissue differentiation and maintenance is ultimately regulated by the coordination between differentiation and proliferation of specific stem cells or progenitors. The importance of these mechanisms has been well documented in early development and in a diversity of organs such as the skin, brain, gut, and hematopoietic system (Fuchs, 2009, Lange and Calegari, 2010, Li and Clevers, 2010). However, the molecular regulations coordinating cell-cycle progression and differentiation remain unclear. The study of such mechanisms with adult stem cells in vivo is challenging for technical reasons, whereas in vitro expansion of these cells remains difficult. Human pluripotent stem cells (hPSCs) generated from embryo at the blastocyst stage (embryonic stem cells or hESCs) or from reprogrammed somatic cells (induced pluripotent stem cells or hIPSCs) represent an advantageous system to model these regulations because they can proliferate indefinitely in vitro while maintaining the capacity to differentiate into the three primary germ layers, neuroectoderm, mesoderm, and endoderm. Furthermore, mechanisms maintaining the pluripotent status of hPSCs and orchestrating their cell fate specification have been broadly studied. Activin/Nodal and FGF cooperate to maintain the expression of pluripotency factors, including Nanog, that in turn block neuroectoderm differentiation (Vallier et al., 2009a, Xu et al., 2008). Accordingly, inhibition of Activin/Nodal signaling induces neuroectoderm differentiation (Chambers et al., 2009). However, Activin/Nodal signaling is also necessary for endoderm differentiation and achieves these apparent contradictory functions by controlling divergent transcriptional networks in pluripotent cells and during endoderm specification (Brown et al., 2011, Mullen et al., 2011). Thus, the activity of Activin/Nodal needs to be tightly controlled in hPSCs and factors influencing this activity can direct their differentiation either toward neuroectoderm or mesendoderm (Chng et al., 2010).

Pluripotency is also linked with cell-cycle regulation because studies in mouse ESCs (mESCs) have shown that their pluripotent status is associated with a specific cell-cycle profile characterized by a shortened G1 phase and the lack of G1 checkpoint regulation (Coronado et al., 2013, Savatier et al., 1996). In conventional cells, cyclin D1–3 are expressed in G1 phase and control the activity of CDK4/6 that inhibit pRB and free E2F to initiate the G1-S transition. In contrast, cyclin Ds are expressed at low level in mESCs, whereas pRB is constitutively phosphorylated by CDK2-cyclin E bypassing the need of a G1 checkpoint (Savatier et al., 1994). hESCs are also characterized by a short G1 phase, whereas their pluripotent status relies on CDK2 activity (Neganova et al., 2009). Furthermore, both hESCs and mESCs display the same resistance to DNA damage suggesting a similar lack in G1 check points (Neganova et al., 2011). However, the mechanisms involved might diverge as indicated by the expression of cyclin D proteins (Neganova et al., 2009) and the presence of normal pRB-cyclin Ds/CDK4/6 cascade in hESCs (Sela et al., 2012). This difference could be explained by species divergence but also by a different embryonic origin, mESCs representing the inner cell mass of preimplantation blastocyst in diapause, whereas hESCs being more similar to epiblast cells of postimplantation embryo (Brons et al., 2007, Tesar et al., 2007). Nevertheless, recent studies have clearly shown that both mouse and human pluripotent stem cells exhibit a lengthening of the G1 phase during differentiation, indicating that differentiation affects cell-cycle regulation and that a truncated G1 phase is a hallmark of the pluripotent state (Calder et al., 2013, Coronado et al., 2013). Besides these observations, the potential interconnections between cell fate choice and cell-cycle progression have remained largely unstudied due to the lack of molecular tools for investigating cell-cycle mechanisms in pluripotent cells. Indeed, cell synchronization using chemical inhibitors in ESCs systematically induces their differentiation, whereas serum deprivation has no effect (Sela et al., 2012).

In this study, we took advantage of the FUCCI (Sakaue-Sawano et al., 2008) reporter system to study the regulation of cell fate choice in hESCs and their capacity to differentiate into chemically defined culture conditions promoting endoderm, mesoderm, and neuroectoderm specification. We observed that hESCs in early G1 phase can only initiate differentiation into endoderm, whereas hESCs in late G1 were limited to neuroectoderm differentiation. Functional experiments reveal that the activity of Activin/Nodal signaling during cell-cycle progression is controlled by cyclin D proteins that activate CDK4/6 and lead to the phosphorylation of Smad2 and Smad3 in their linker region. This mechanism blocks Smad2/3 shuttling in the nucleus in late G1, thereby preventing endoderm and allowing neuroectoderm specification. Thus, cyclin D proteins act as essential regulators of signaling pathways controlling early cell fate decisions. These results unravel the molecular mechanisms by which cell fate decisions are controlled by the cell-cycle machinery and uncover how self-renewal and pluripotency could be coordinated.

## Results

### Generation of FUCCI-hESC Reporter Lines for Studying Cell-Cycle Progression

**Figure 1:**
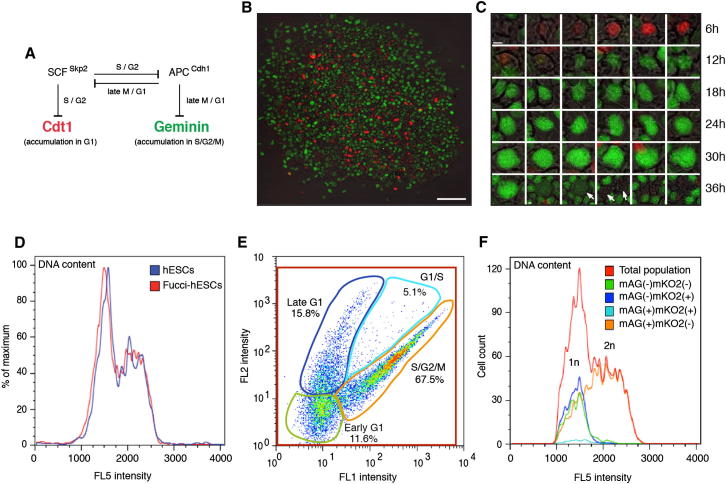
Generating Fucci-hESCs for Studying Cell-Cycle-Dependent Events in Live Pluripotent Stem Cells (A) Mechanistic overview of the FUCCI system. FUCCI system relies on the fusion of a red and green fluorescent protein (red mKO2 and green mAG) to two cell-cycle-specific proteins (Cdt1 and Geminin). (B) Representative colony of FUCCI-hESCs showing cells in early G1 (no fluorescence), late G1 (red), G1/S transition (yellow), and S/G2/M (green). Scale bar represents 100 μm. (C) Time-lapse imaging of a FUCCI-hESC progressing through the cell cycle. Early G1 cells express neither red mKO2-Cdt1 nor green mAG-Geminin. Late G1 cells express red mKO2-Cdt1. mAG-Geminin starts being expressed in G1/S transition giving the cells a temporal yellow color due to coexpression with mKO2-Cdt1. Cells in S, G2, and M phase expressing only the green mAG-Geminin. During cell division, mAG-Geminin is rapidly degraded and the resulting daughter cells are not fluorescent (arrows). Scale bar represents 5 μm. (D) FUCCI system does not alter the cell-cycle distribution of hESCs. H9 hESCs and FUCCI-hESCs were analyzed for DNA content by flow cytometry and Hoechst staining. (E) Analysis of the relative proportion of FUCCI-hESCs in each cell-cycle phase. FUCCI-hESCs were analyzed by flow cytometry for mAG-Geminin (FL1) and mKO2-Cdt1 (FL2) expression. (F) DNA content analysis of different cell-cycle phases in FUCCI-hESCs. FUCCI-hESCs were stained with Hoechst and subpopulations of cells were gated as shown in (E). Green, early G1 phase; blue, late G1 phase; light blue, G1/S transition; orange, S/G2/M phase; red, total population.

**Figure S1:**
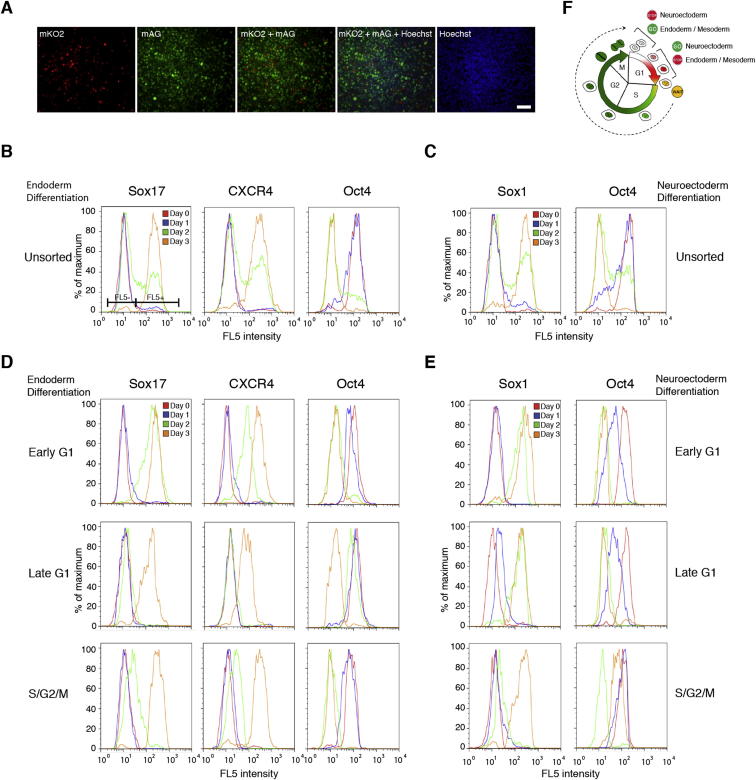
Generating FUCCI-hESCs for Studying Cell-Cycle-Dependent Events in Live Pluripotent Stem Cells, Related to Figure 2 (A) Representative colony of Fucci-hESCs. Fucci-hESCs fixed with 4% PFA and visualized by fluorescence microscopy. Scale bar, 100 μm. (B–E) hESCs differentiate nonsynchronously and nonhomogenously. Live unsorted Fucci-hESCs were differentiated into (B) endoderm and (C) neuroectoderm and analyzed for germ layer and pluripotency marker expression at different time points flow cytometry. Gates depict negative FL5- and positive FL5+ populations. (D-E) Early G1 phase directs endoderm whereas late G1 promotes neuroectoderm differentiation. Live Fucci-hESCs sorted into early G1 phase, late G1, G1/S transition or S/G2/M phase cells were differentiated into (D) endoderm or (E) neuroectoderm and analyzed for germ layer and pluripotency marker expression at different time points by flow cytometry. (F) Overview of the initiation of differentiation in hESCs. Early G1 phase directs cells into endoderm and mesoderm whereas neuroectoderm is blocked. Late G1 phase directs cells into neuroectoderm whereas endoderm and mesoderm is blocked.

Analysis of cell-cycle-specific events in pluripotent cells is challenging because cell-cycle synchronization using chemicals induces their differentiation (data not shown). To overcome this limitation, we decided to adapt the FUCCI reporter system to hESCs (Figure 1A) grown in fully defined culture conditions (Vallier et al., 2005). We first subcloned the *Geminin-mAG* and *Cdt-mKO2* fusion genes in the pTP6 system that enables stable transgene expression based on Puromycin or Neomycin resistance (Vallier et al., 2004). The resulting vectors were cotransfected in hESCs using Lipofectamine 2000 as described previously (Vallier et al., 2004). Following antibiotics selection, we picked individual hESC colonies containing green, red, and yellow fluorescent cells for further analyses (Figure 1B and Figure S1A available online). Of note, the resulting subclonal FUCCI-hESCs were grown in the presence of Puromycin and Neomycin to guarantee constant and homogenous expression of the reporter genes.

We first performed time-lapse microscopy on single FUCCI-hESC and observed the expected transition from red to green fluorescence upon progression of the cell cycle (Figure 1C). Furthermore, fluorescence-activated cell sorting (FACS) analyses combined with Hoechst staining showed that FUCCI-hESCs have the same cell-cycle profile as nontransfected hESCs (Figure 1D) demonstrating that transgene overexpression did not alter cell-cycle regulation. These analyses also confirm that the fluorescent proteins were expressed as expected during the cell cycle (Figures 1E and 1F: green fluorescent cells in G2, nonfluorescent cells in early G1, red fluorescent in late G1, and yellow fluorescent cells in G1/S). Furthermore, FACS analyses showed that sorted FUCCI-hESCs express pluripotency factors homogenously (95% Oct-4+ cells) independently of their cell cycle, whereas differentiation markers such as Sox1 (<1%) or Sox17 (<4%) were expressed in a small fraction of cells distributed equally in the different phases of the cell cycle (Figures S1D and S1E; data not shown). Similar results have been obtained with Nanog, Tra-1-60, Eomes, Mixl1, Goosecoid, and Pax6 (data not shown), confirming that hESCs in our culture system grow as a near-homogenous population with a minimal background of differentiation. Considered collectively, these results demonstrate the efficacy of the FUCCI reporter system to sort hESCs into different phases of their cycle without altering their fundamental characteristics.

### Differentiation Capacity of hESCs Varies during Progression of the G1 Phase

**Figure 2:**
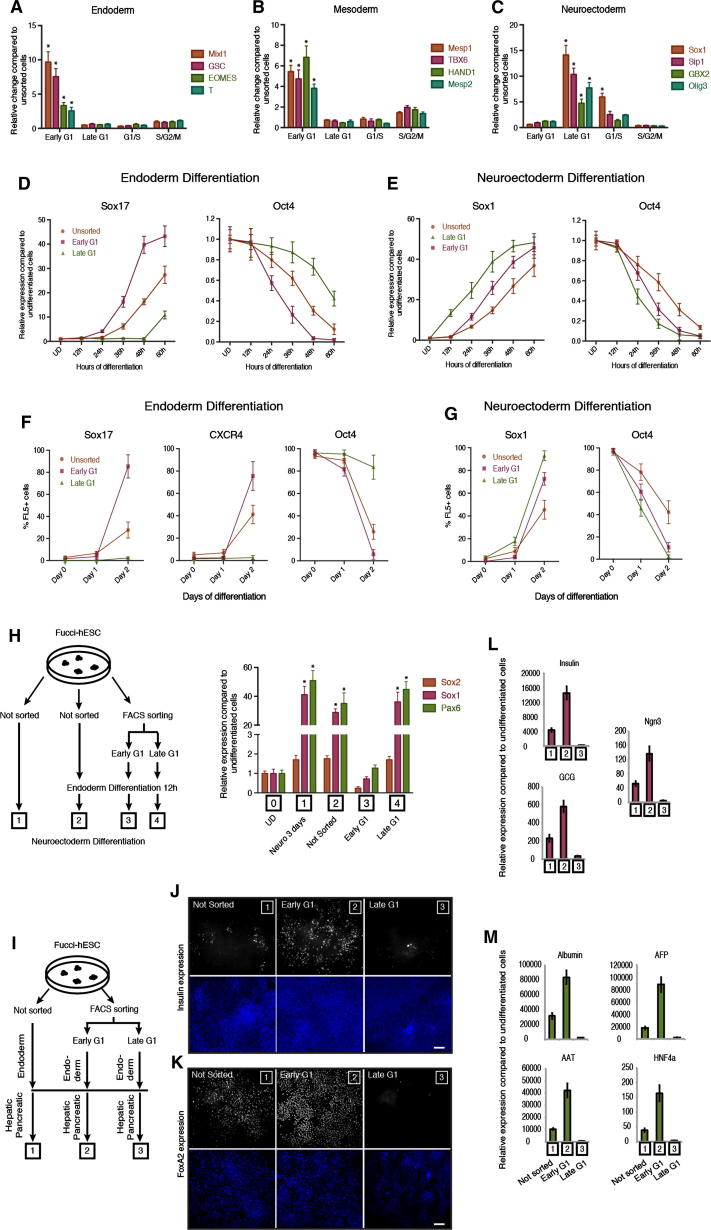
The Cell Cycle Directs Differentiation of hESCs (A–C) Cell-cycle-dependent differentiation of hESCs. qPCR analysis for the expression of germ layer markers in FACS sorted Tra-1-60-positive FUCCI-hESCs incubated for 6 hr in culture condition inductive for three germ layers. (D and E) Cell cycle regulates the timing of differentiation in hESCs. Fucci-hESCs sorted into early G1 phase, late G1, G1/S transition, or S/G2/M phase cells were differentiated into (D) endoderm or (E) neuroectoderm and analyzed for germ layer and pluripotency marker expression at different time points by qPCR. (F and G) Early G1 phase directs endoderm whereas late G1 promotes neuroectoderm differentiation. Flow cytometry analysis for the expression of germ layer markers in FACS sorted Fucci-hESCs incubated for up to two days in culture condition inductive for (F) endoderm or (G) neuroectoderm differentiation. (H) Restricted capacity of differentiation during G1 transition. Left: schematic overview of experimental approach. Right: qPCR analysis of neuroectoderm markers in samples (1–4) treated as shown in schematic overview. (I) Schematic presentation of pancreatic differentiation from sorted or unsorted cells. (J) Cell-cycle stage of pluripotent cells affects insulin expression during pancreatic differentiation. Immunostaining of insulin during pancreatic differentiation. (K) Cell-cycle stage affects foregut marker FoxA2 expression. Immunostaining for FoxA2 at day 8 of hepatic differentiation of hESCs sorted in early G1 or late G1 phase. (L) Cell-cycle stage of pluripotent cells affects pancreatic differentiation. Early G1 phase cells differentiating into endoderm improves pancreatic differentiation, whereas late G1 phase cells reduces pancreatic differentiation. (M) Cell-cycle stage affects liver differentiation. Expression of liver markers at day 25 of hepatic differentiation shows variability of developmental potential for cells that were used during initial endoderm differentiation. All data are shown as mean ± SD (n = 3). Student’s t test was performed. ^∗^p < 0.05. Scale bar represents 100 μm. See also Figure S1.

Previous studies have shown that cell fate decisions could be made during the G1 phase (Calder et al., 2013, Sela et al., 2012). To investigate this possibility in more detail, we examined the capacity of hESCs to respond to differentiation signals at different phases of the cell cycle. For that, Tra-1-60-positive FUCCI-hESCs were sorted according to their fluorescence and then placed in defined culture conditions driving differentiation into homogenous populations of endoderm, mesoderm and neuroectoderm cells (Vallier et al., 2009b, Vallier et al., 2009c). This analysis revealed that hESCs in early G1 could only initiate endoderm/mesoderm gene expression (see Mixl1, Goosecoid, Eomes, T, Mesp1, TBX6, HAND1, Mesp2; Figures 2A and 2B; Table S1), whereas hESCs in late G1 could only initiate the expression of neuroectoderm markers (see Sox1, Sip1, Gbx2, Olig3; Figure 2C). On the other hand, cells in G2/S/M phases of the cell cycle responded poorly to differentiation signals (Figures 2A–2C). These results confirm that induction of differentiation in hESCs occurs during the G1 phase of the cell cycle but also reveal that hESCs in early and late G1 might have a different capacity of differentiation.

Because these experiments represent an early snapshot of gene transcription after only 6 hr of differentiation, we decided to investigate the capacity of sorted FUCCI-hESCs to differentiate into neuroectoderm and endoderm over a prolonged period of time. FACS and quantitative PCR (qPCR) analyses showed that early G1 hESCs differentiated into a near-homogenous population of endoderm cells (85% Sox17+/80% CXCR4+/3% Oct4+; Figures 2D, 2F, and S1D) in 48 hr, whereas unsorted hESCs (35% Sox17+/40% CXCR4+/23% Oct4+; Figures 2D, 2F and S1B) or late G1-hESCs (5% Sox17/4% CXCR4+/80% Oct4; Figures 2D, 2F, and S1B) grown in the same conditions remained a heterogeneous population of cells. The opposite results were obtained for neuroectoderm differentiation (Figures 2E, 2G, S1C, and S1E). These observations confirmed that hESCs display different capacity of differentiation upon the progression of the G1 phase. Early G1-hESCs are permissive for initiating endoderm specification, whereas late G1-hESCs are permissive for neuroectoderm differentiation (Figure S1F).

### The Efficiency of hESCs to Differentiate into Tissue-Specific Cells Is Influenced by Their Cell Cycle

The results described above do not exclude the possibility to generate homogenous populations of differentiated cells from hESCs as we and others have shown (Vallier et al., 2009b). Indeed, hESCs grown in inductive culture conditions are constantly cycling and thus sooner or later receive signals initiating their differentiation. Accordingly, unsorted FUCCI-hESCs generate populations of endoderm cells expressing homogenously Sox17/CXCR4 (95% + cells; data not shown) after 80 hr of differentiation. However, our observations could explain why differentiation of hESCs is always asynchronous with cells upregulating differentiation markers more rapidly than others (Figures 2D, 2F and S1B). Indeed, a late G1-hESC will delay the initiation of its differentiation toward the endoderm lineage for an additional cell cycle (∼30 hr) before reaching the next early G1 phase. This mechanism would result in a population of cells differentiating toward the same lineage but at varying developmental stages. This hypothesis also implies that a cell in a specific phase of the cell cycle could lose its capacity to differentiate toward other lineages more rapidly.

To validate this point, Tra-1-60-positive FUCCI-hESCs sorted into early G1 or late G1 were grown in conditions inductive for endoderm differentiation for 12 hr and then for an additional 72 hr in conditions inductive for neuroectoderm differentiation (Figure 2H). qPCR analyses showed that early G1 hESCs induced to differentiate toward endoderm lose their capacity to express neuroectoderm markers contrary to late G1 hESCs grown in similar culture conditions (Figure 2H). These results confirmed that early G1-hESCs can differentiate more rapidly into endoderm than cells in late G1 and validate in part our hypothesis concerning the link between asynchronous differentiation of pluripotent cells and their cell-cycle state. These results also suggest that hESCs synchronized in early G1 could differentiate more efficiently into endoderm derivatives. This prompted us to investigate the importance of the cell cycle for the production of terminally differentiated cells such as pancreatic and hepatic cells. hESCs synchronized in early G1 or late G1 were differentiated into both cell types using defined culture conditions as described previously (Cho et al., 2012). qPCR and immunostaining analyses showed that early G1-hESCs were able to differentiate more efficiently into insulin-expressing cells and hepatocyte-like cells when compared to unsorted cells or cells sorted in late G1 as shown by the increase in the number of c-peptide-positive cells and in the expression of endocrine markers for pancreatic differentiation (Ngn3, Insulin, and Glucagon; Figures 2J and 2L) and the increase in the expression of hepatocytes markers for hepatic differentiation (FoxA2, Albumin, AFP, HNF4a, and A1AT; Figures 2K and 2M). Taken together, these results demonstrate that the phase of the cell cycle during which hESCs initiate their differentiation can influence their capacity to generate differentiated cells and underlines the importance of cell-cycle regulation in the mechanisms controlling cell fate decisions.

### Cyclin Ds Are Necessary to Maintain Pluripotency of hESCs by Preventing Endoderm Differentiation Induced by Activin/Nodal Signaling

**Figure 3:**
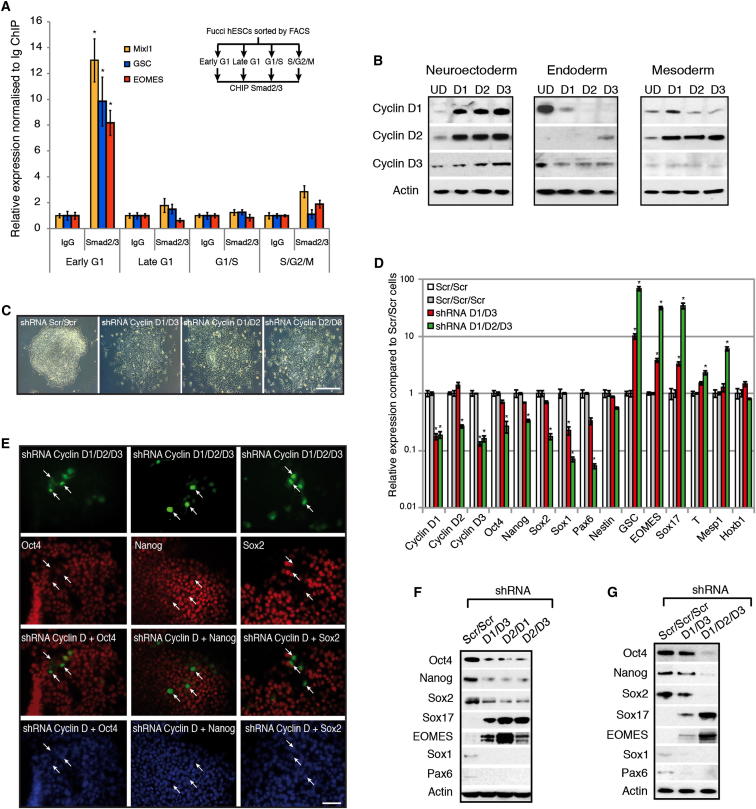
Cyclin Ds Are Necessary for Pluripotency (A) Cell-cycle-dependent binding of Smad2/3 to endoderm genes. ChIP analyses in Tra-1-60+ sorted FUCCI-hESCs showing Smad2/3 binding on endoderm genes. (B) Cyclin D expression during early differentiation of hESCs. Cyclin D1-3 protein expression during days 1–3 of neuroectoderm, endoderm, and mesoderm differentiation shown by western blot analysis. (C) Morphology of cyclin D double knockdown. Representative colonies of shRNA Scramble and cyclin D double-knockdown cells. (D) Triple knockdown of cyclin D causes endoderm differentiation. cyclin D1/3 double-knockdown cells were transfected with a cyclin D2 shRNA construct expressing GFP and then FACS sorted for qPCR analyses. (E) Triple knockdown of cyclin D causes loss of pluripotency markers. Immunofluorescence microscopy analyses for Oct4, Nanog, and Sox2 expression (red) in cyclin D triple-knockdown hESCs (green / arrows). (F) Double knockdown of cyclin D causes endoderm differentiation and blocks neuroectoderm differentiation. Cyclin D double-knockdown cells were analyzed for germ layer marker expression by western blot. (G) Triple knockdown of cyclin D causes endoderm differentiation. Cyclin D1/3 double-knockdown cells were transfected with a cyclin D2 shRNA construct expressing GFP and then FACS sorted for western blot analyses. UD, undifferentiated cells. Student’s t test was performed. ^∗^p < 0.05. Scale bar represents 100 μm. See also Figures S2 and S3.

**Figure S2:**
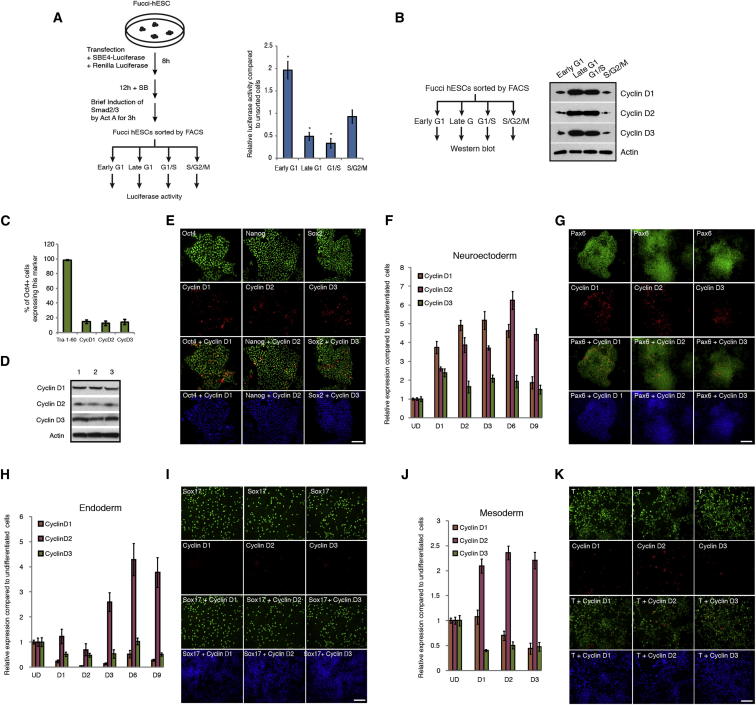
Cyclin D Expression in hESCs and during Germ Layer Specification, Related to Figure 3 (A) Smad2/3 transcriptional activity during progression of the cell cycle. Fucci-hESCs transfected with a reporter for Smad2/3 transcriptional activity (SBE4-luciferase construct) were incubated with Activin A for 3h followed by FACS sorting and analysis of luciferase activity. (B) Cyclin D expression during cell cycle progression in hESCs. Western blot analysis for cyclin D1-D3 expression in FACS sorted Fucci-hESCs. (C) Cyclin D expression in hESCs. Flow cytometry analysis of cyclin D expression in Oct4+ pluripotent cells. (D) Cyclin D expression in hESCs is stable. hESCs were collected at day 3 after three days of splittings and analyzed by western blot. (E) Cyclin D expression in pluripotent cells. Immunofluorescence microscopy of pluripotency markers and cyclin D1-3 in hESCs. (F–K) Cyclin D expression during differentiation of hESCs into neuroectoderm (F, G), endoderm (H, I) and mesoderm (J, K) by Q-PCR and immunostaining, respectively. UD - undifferentiated H9; D1-D9 day 1 to 9. Data shown as mean ± SD (n = 3). Scale bar, 100μm.

The capacity of hESCs to initiate their differentiation toward the endoderm lineage during the early G1 phase suggest that signaling pathways inducing this differentiation might be more active during this phase of the cell cycle. Activin/Nodal signaling has been shown to be the main inducer of endoderm differentiation (D’Amour et al., 2005). Therefore, we decided to define the transcriptional activity of Activin/Nodal-Smad2/3 signaling during cell-cycle progression of hESCs. We first performed Smad2/3 chromatin immunoprecipitation (ChIP) on Tra-1-60-positive sorted Fucci-hESCs and observed that Smad2/3 bound specifically to endoderm genes in early G1 (Figure 3A; Table S2) and not in any other part of the cell cycle. Furthermore, transfection of Fucci-hESCs with a luciferase reporter for Smad2/3 transcriptional activity confirmed that the Activin/Nodal-Smad2/3 pathway is less potent during the late G1 phase and G1/S transition (Figure S2A). These results show that the activity of the Activin/Nodal signaling pathway is cell-cycle dependent and suggest the existence of mechanisms controlling activity/binding of Smad2/3 during cell-cycle progression.

Because Smad2/3 binding to endoderm loci was blocked during the change from early to late G1 phase, we hypothesized that factors specifically expressed during this cell-cycle phase could control Activin/Nodal-Smad2/3 pathway. Cyclin D proteins represented interesting candidates because they are central for coordinating G1 progression in conjunction with their catalytic partners CDK4/6. Furthermore, cyclin D1–3 proteins are expressed in hESCs (Figures S2C–S2E; Table S3) as shown by others (Neganova et al., 2009) and exhibit a cell-cycle-dependent expression in hESCs peaking at late G1 and G1/S phase (Figure S2B) when transcriptional activity of Activin/Nodal signaling pathway is diminished (Figure S2A). Therefore, we decided to study the pattern of cyclin D expression during germ layer specification in vitro. Western blot and qPCR analyses showed that differentiation of hESCs toward neuroectoderm resulted in a rapid induction of all three cyclin Ds (Figures 3B, S2F, and S2G), whereas endoderm differentiation (Figures 3B, S2H, and S2I) was accompanied by a decrease in cyclin D1 and low expression of cyclin D2/D3. Mesoderm differentiation showed an upregulation of cyclin D2, whereas cyclin D1 and D3 exhibited a minor decrease (Figures 3B, S2J, and S2K). Thus, cyclin Ds are highly expressed during lineage specification that require Activin/Nodal signaling inhibition (neuroectoderm) whereas their expression is lower during specification of lineages requiring Activin/Nodal signaling activity (endoderm and mesoderm). Together, these data demonstrate that each germ layer is associated with a specific level/pattern of cyclin D expression.

**Figure S3:**
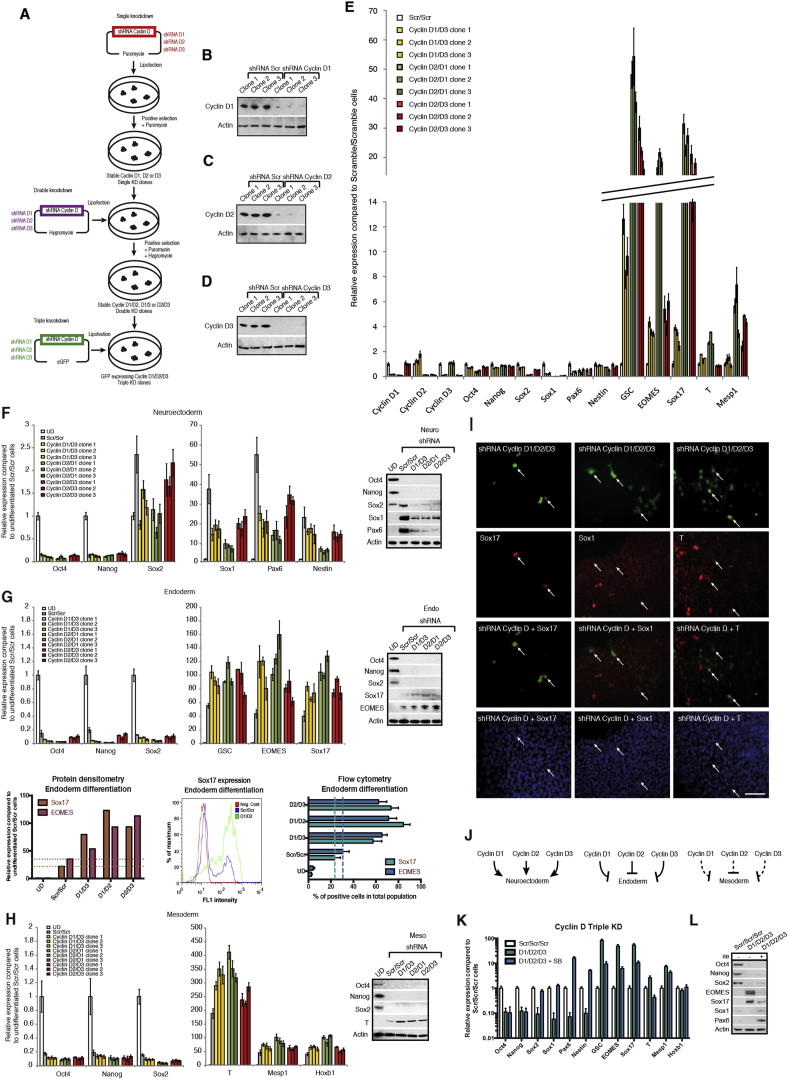
Cyclin D Triple Knockdown Causes Loss of Pluripotency, Related to Figure 3 and Table S4 (A) Schematic overview of cyclin D knockdown cell line generation. (B–D) Cyclin D expression in knockdown clones compared to cells transfected with a Scramble shRNA expression vector. Expression of (B) cyclin D1, (C) cyclin D2, (D) cyclin D3 in corresponding knockdown clones. (E) Double knockdown of cyclin D1-D3 decrease neuroectoderm markers and increase endoderm/mesoderm markers. Cyclin D double knockdown cells were analyzed by Q-PCR. (F–H) Differentiation of cyclin D double knockdown cells. Cyclin D double knockdown cells were differentiated into (F) Neuroectoderm, (G) Endoderm or (H) Mesoderm and expression of germ layer markers was analyzed by Q-PCR or western blot. Data are normalized to undifferentiated cells (UD). (G bottom left panel) Protein quantification of western blots by densitometry. (G bottom middle panel) Representative data from endoderm differentiation of cyclin D double knockdown cells. Cells were analyzed by flow cytometry after 3 days of endoderm differentiation. Red – negative control, Blue – Scramble/Scramble cells, Green – Double knockdown of cyclin D1 and cyclin D2. (G bottom right panel) Cyclin D double knockdown increases endoderm differentiation. Cells were analyzed by flow cytometry after 3 days of differentiation. (I) Triple knockdown of cyclin D causes endoderm differentiation. Immunofluorescence microscopy of Sox17, Sox1 and T (red) in cyclin D1/3 double knockdown cells transfected with a cyclin D2 shRNA construct expressing GFP (green). Triple knockdown cells overlap only with Sox17 positive cells (arrows). (J) Schematic representation of cyclin D1-3 function during early differentiation. Cyclin D proteins promote neuroectoderm differentiation while strongly reducing endoderm differentiation and moderately reducing mesoderm differentiation. (K and L) Expression of endoderm markers in cyclin D triple knockdown cells depends on Activin signaling. Triple knockdown cells were treated with Activin/Nodal signaling inhibitor SB-431542 for 48 hr and analyzed by (K) Q-PCR or (L) western blot for marker expression. Scale bar, 100μm. Data shown as mean ± SD (n = 3).

These observations prompted us to study the function of cyclin D proteins in hESCs. For that, we stably knocked down the expression of the three cyclin Ds in all possible combinations (Figures S3A–S3D). Single-knockdown hESC lines (ShD1-, ShD2-, and ShD3-hESCs) were able to self-renew, although we observed a moderate increase in expression of differentiation markers, especially mesoderm/endoderm genes (data not shown). Double-knockdown hESCs (ShD1D2, ShD2D3, and ShD1D3-hESCs) showed a propensity for spontaneous differentiation into cells expressing endoderm markers, whereas pluripotency and neuroectoderm marker expression was systematically diminished (Figures 3C, 3D, 3F, and S3E). Furthermore, double-knockdown hESCs displayed a diminished capacity to differentiate into neuroectoderm and an increased capacity to differentiate into endoderm/mesoderm (Figures S3F–S3H; Table S4). Finally, triple-knockdown hESCs (shD1D2D3-hESCs) could not be expanded in vitro suggesting an essential function for cyclin Ds in pluripotency and/or self-renewal. To bypass this limitation, GFP-expressing knockdown constructs were transitory transfected into cyclin D double-knockdown cells showing that decreased expression of all three cyclin Ds resulted in the loss of pluripotency markers while inducing differentiation into endoderm (Figures 3D, 3E, 3G, and S3I–S3L). Taken together, these data demonstrate that cyclin Ds are necessary to maintain pluripotency in hESCs by limiting their capacity to differentiate into endoderm.

**Figure 4:**
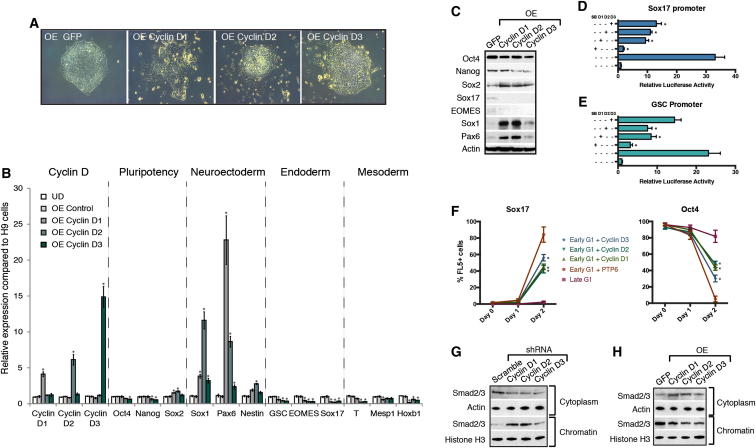
Cyclin D Overexpression in hESCs Induces Neuroectoderm Differentiation (A) Morphology of cyclin D overexpression (OE). Representative colonies of GFP OE and cyclin D OE hESCs. (B and C) Cyclin D OE overexpression causes neuroectoderm differentiation and decreases endoderm markers. Expression of neuroectoderm markers in cyclin D overexpressing cells shown by qPCR (B) or western blot (C). (D and E) Cyclin Ds repress endoderm loci. Luciferase constructs with Sox17 (D) or GSC (E) promoter regions containing Smad2/3 binding sites were cotransfected with cyclin D OE constructs, then differentiated into endoderm for 48 hr and analyzed for luciferase activity. (F) Cyclin Ds repress the initiation of endoderm differentiation in early G1 phase. Fucci-hESCs transfected with cyclin D OE constructs were sorted into early G1 phase and analyzed for marker expression by flow cytometry after endoderm differentiation. (G) Cyclin D knockdown causes the accumulation of Smad2/3 on chromatin. Relative amount of Smad2/3 protein in cytoplasm and on chromatin in cyclin D1-3 knockdown cells compared to Scramble shRNA overexpressing cells. (H) Cyclin D overexpression results in Smad2/3 accumulation in the cytoplasm. Smad2/3 localization in cytoplasm and on chromatin was analyzed in cyclin D1, D2, and D3 overexpressing cells by western blot. All data are shown as mean ± SD. (n = 3). Student’s t test was performed. ^∗^p < 0.05. See also Figure S4.

**Figure S4:**
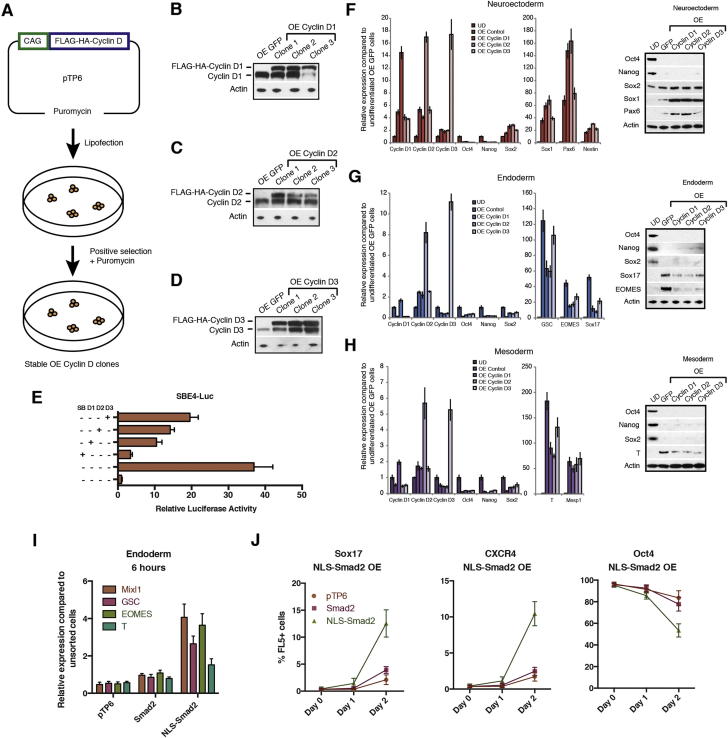
Cyclin D Overexpression Induces Neuroectoderm and Blocks Endoderm/Mesoderm, Related to Figure 4 (A) Schematic overview of the approach used to generate cyclin D overexpression (OE) hESC lines. (B–D) Cyclin D overexpression clones. Cyclin D expression in (B) cyclin D1 OE clones, (C) cyclin D2 OE clones or (D) cyclin D3 OE clones compared to OE GFP. All OE cyclin D1, D2 and D3 clones showed a constitutive expression of cyclin D proteins that were similar to endogenous cyclin D levels, thus resembling physiological conditions. (E) Cyclin Ds repress Smad2/3 dependent transcription. H9 cells cotransfected with SBE4-Luc and cyclin D constructs were analyzed for luciferase signal after 48 hr of transfection. (F–H) Overexpression of cyclin D1-D3 results in increased neuroectoderm markers and decreased endoderm/mesoderm markers. Cyclin D OE cells were differentiated into (F) Neuroectoderm, (G) Endoderm or (H) Mesoderm and differentiation markers were analyzed by Q-PCR or western blot. (I and J) Constitutively nuclear Smad2 initiates endoderm differentiation in late G1 cells. Fucci-hESCs transfected with Smad2 constructs were sorted into late G1 phase and analyzed for marker expression by (I) Q-PCR after 6 hr or (J) by flow cytometry 1-2 days after endoderm differentiation. Data are normalized to undifferentiated cells (UD). Scale bar, 100 μm. Data shown as mean ± SD (n = 3).

We then performed the opposite experiments by stably overexpressing cyclin Ds in hESCs (Figures 4A and S4A–S4D). The resulting hESCs (OED-hESCs) maintained self-renewal and pluripotency, but showed an increase in neuroectoderm marker expression (Figures 4B and 4C). Furthermore, cyclin OED-hESCs display an enhanced capacity to differentiate into neuroectoderm (Figure S4F) and have a limited capacity to differentiate into mesoderm/endoderm (Figures S4G and S4H). Collectively, these gain of function experiments show that cyclin Ds promote neuroectoderm differentiation while being able to inhibit endoderm differentiation induced by Activin/Nodal signaling. Therefore, cyclin Ds could inhibit the activity associated with Activin/Nodal signaling especially in late G1 when they are highly expressed.

### Cyclin Ds Control the Transcriptional Activity of Smad2/3

**Figure 5:**
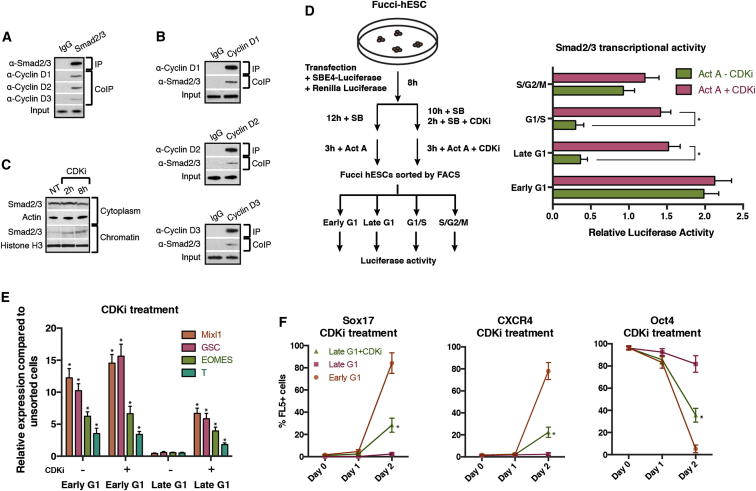
Cyclin D/CDK4/6 Control Smad2/3 Transcriptional Activity (A) Smad2/3 interacts with cyclin D proteins. Smad2/3 was immunoprecipitated and analyzed for the presence of cyclin D1–3 by western blot. (B) Cyclin D proteins interact with Smad2/3. Cyclin D1–3 were immunoprecipitated and analyzed for the presence of Smad2/3 by western blot. (C) CDK4/6 inhibition by small molecule results in Smad2/3 accumulation on chromatin. hESCs cells were treated with CDK4/6 inhibitor (CDKi or 0.75 μM PD0332991) for 2 hr or 8 hr and then Smad2/3 localization in cytoplasm and on chromatin was analyzed using western blot. (D) Smad2/3 transcriptional activity is repressed in late G1 phase by CDK4/6. Left: schematic overview of the experiment. Right: FUCCI-hESCs were transfected with a Smad2/3-dependent Luciferase expression construct and incubated with Activin A in the presence or absence of 0.75 μM PD0332991. (E and F) CDK4/6 inhibition partially removes the endoderm differentiation blockage from late G1 phase cells. Sorted FUCCI-hESCs were differentiated into endoderm in the presence or absence of 0.75 μM PD0332991 and analyzed by qPCR (E) after 6 hr or flow cytometry (F) after 1–2 days of endoderm differentiation. Student’s t test was performed. ^∗^p < 0.05.

**Figure S5:**
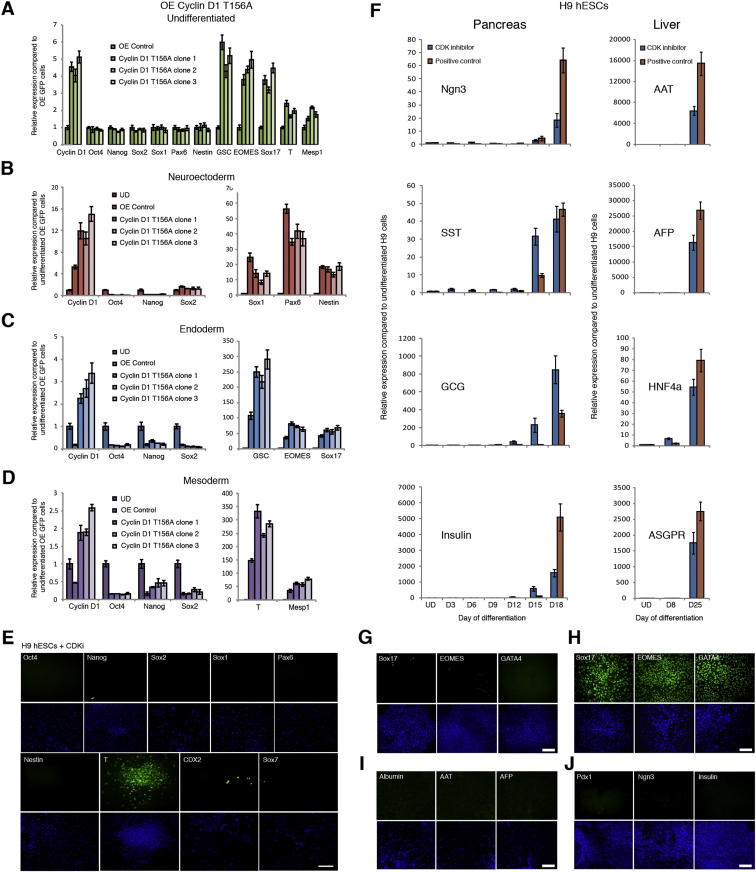
CDK4/6 Inhibition in hESCs Causes Endoderm Differentiation, Related to Figure 7 (A–D) Cyclin D1 T156A mutant increases endoderm/mesoderm and decreases neuroectoderm differentiation of hESCs. (A) Cyclin D1 T156A overexpressing cells were analyzed by Q-PCR for marker expression or differentiated into (B) Neuroectoderm, (C) Endoderm or (D) Mesoderm and then analyzed by Q-PCR. Data are normalized to undifferentiated cells (UD). Data shown as mean ± SD (n = 3). (E) CDK4/6 inhibition in H9 cell line. hESCs were grown in the presence of CDK4/6 inhibitor 0.75μM PD0332991 for 6 days and analyzed for germ layer marker expression by immunostaining. (F) CDKi-produced endoderm can give rise to pancreatic and hepatic cells. H9 hESCs were differentiated into endoderm with 0.75μM PD0332991 for 6 days and then the resulting cells were grown in culture conditions inducing pancreatic and hepatic differentiation for 12 and 22 days respectively. Marker expression was analyzed by Q-PCR. Conventional endoderm differentiation protocol was used as a positive control for pancreatic and hepatic differentiation. Data shown as mean ± SD (n = 3). (G and H) Immunostaining of negative control cells cultured in the absence of CDKi (G) or positive control cells differentiated with the standard endoderm protocol and stained for endoderm markers (H). (I and J) Immunostaining of negative control cells for liver (I) or pancreatic (J) differentiation. Cells were first cultured in the absence of CDKi and then differentiated into hepatocytes or pancreatic cells and immunostained for the corresponding markers.

We then decided to further investigate the molecular mechanisms by which cyclin D could control Activin/Nodal signaling. Smad2/3 act as main effectors of this pathway and thus represent obvious candidates for this kind of regulation. Accordingly, we observed that cyclin D overexpression decreased Smad2/3 transcriptional activity in hESCs (Figure S4E) and in endoderm cells (Figures 4D and 4E). Constitutive overexpression of cyclin Ds also decreased the propensity of early G1 hESCs to initiate endoderm differentiation (Figure 4F) and reduced the number of early G1 phase cells (11.7% in PTP6, 5.8% in OE cyclin D1, 6.3% in OE cyclin D2, and 7.5% in OE cyclin D3) while increasing late G1 phase cells (15.8% in PTP6, 18.6% in OE cyclin D1, 17.9% in OE cyclin D2, and 17.2% in OE cyclin D3). Finally, western blot analyses revealed that knockdown in cyclin D expression resulted in an increase in Smad2/3 proteins localized on chromatin, whereas overexpression of cyclin D had the opposite effect (Figures 4G and 4H). Therefore, the level of cyclin D proteins appears to modulate the shuttling of Smad2/3 in and out of the nucleus. To validate these observations, late G1-hESCs were transitorily transfected with a Smad2 protein containing a nuclear localization signal (NLS) (Lo and Massagué, 1999) and the resulting cells were induced to differentiate into endoderm. qPCR analyses 6 hr after sorting (Figure S4I) or flow cytometry after 24–48 hr of differentiation (Figure S4J) show that this constitutive nuclear Smad2 was able to induce the expression of endoderm markers in late G1 hESCs thereby reinforcing the hypothesis that the cell fate restriction observed during cell-cycle progression could be associated with Smad2/3 cellular localization. Importantly, coimmunoprecipitation analyses showed that cyclin D1/D2/D3 interact with Smad2/3 in hESCs (Figures 5A and 5B) suggesting that nuclear transport of Smad2/3 could be controlled by this interaction. Interestingly, a previous study in a cancer cell line has shown that CDK4/6 could control the transcriptional activity of Smad2/3 (Matsuura et al., 2004). In accordance, overexpression of a mutant form of cyclin D1 (T156A) known to inhibit the catalytic activity of CDK4/6 (Diehl and Sherr, 1997) increase the expression of endoderm and mesoderm markers in hESCs (Figure S5A). Furthermore, the same cells display an increased capacity to differentiate into mesoderm/endoderm and a decreased capacity to express neuroectoderm markers (Figures S5B–S5D) when grown in the corresponding inductive conditions. Together, these results suggest that cyclin D-CDK4/6 inhibit the transcriptional activity of Activin signaling by controlling the cellular localization of Smad2/3.

**Figure 6:**
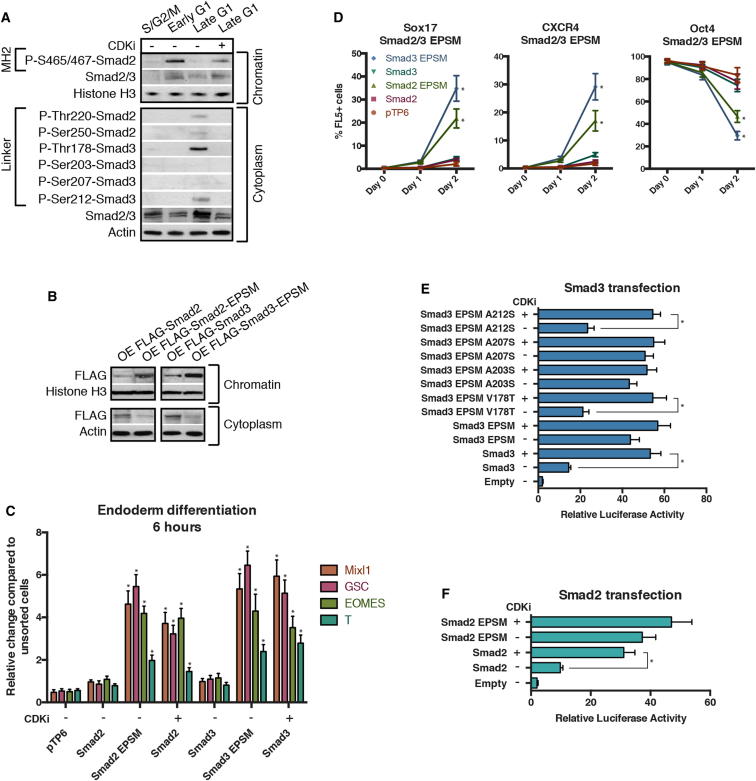
Cyclin D-CDK4/6 Regulates Smad2/3 Shuttling in hPSCs by Linker Phosphorylation (A) Smad2/3 intracellular localization and phosphorylation during cell-cycle progression depends on CDK4/6. Cytoplasm and chromatin were isolated from sorted Fucci-hESCs and analyzed by western blot. (B) Smad2/3 phosphorylation by CDK4/6 regulates Smad2/3 localization to chromatin. Cytoplasmic and chromatin fractions were isolated from H9 cells 48 hr after transfection with Flag-tagged Smad2/3 constructs. (C and D) Sorted Smad2/3 linker phosphorylation mutants can initiate endoderm in late G1 phase. Fucci-hESCs transfected with Smad2/3 constructs were sorted into late G1 cells, and analyzed by qPCR after 6 hr of endoderm differentiation (C) or flow cytometry after 1–2 days of endoderm differentiation (D). (E and F) Smad2/3 phosphorylation in linker residues by CDK4/6 blocks Smad2/3 transcriptional activity. FUCCI-hESCs were cotransfected with SBE4-Luc construct together with Smad3 mutant constructs (E) or Smad2 mutant constructs (F), sorted after 48 hr into late G1 phase and analyzed for luciferase activity. CDK4/6 was inhibited by 0.75 μM PD0332991 for 6 hr prior to analysis. Student’s t test was performed. ^∗^p < 0.05.

To further investigate the molecular mechanisms involved in this regulation, we specifically inhibited CDK4/6 in hESCs using the small molecule PD0332991 (Fry et al., 2004) and observed an increase in Smad2/3 localization to the chromatin fraction (Figure 5C). Inhibition of CDK4/6 in late G1 phase also resulted in an increase in Smad2/3-dependent transcription (Figure 5D) and partially removed the mechanism blocking endoderm differentiation in late G1 (Figures 5E and 5F). Taken together, these data suggest that cyclin D/CDK activity could regulate endoderm specification by controlling the transport of Smad2/3 into the nucleus. Because Smad2/3 nuclear shuttling is mediated by posttranslational modifications (Kretzschmar et al., 1999), we investigated the subcellular localization and phosphorylation status of Smad2 and Smad3 during cell-cycle progression (Figure 6A). Western blot showed that Smad2 and Smad3 are both specifically phosphorylated in their linker region in late G1 when they were excluded from the chromatin (Figure 6A). On the other hand, Smad2/3 proteins were phosphorylated in their MH2 region (P-S465/467) in early G1 when they were more abundant on the chromatin. Moreover, inhibition of CDK4/6 activity resulted in the loss of Smad2/3 linker region phosphorylation and accumulation of Smad2/3 onto the chromatin (Figure 6A). Consequently, CDK4/6 appear to regulate Smad2/3 nuclear shuttling in late G1 of hESCs by phosphorylating specific residues of their linker regions. To confirm these observations, we overexpressed in late G1 hESCs mutant forms of Smad2/3 devoid of phosphorylation sites in the linker region (Smad2-EPSM: T220V, S245A, S250A, S250A; Smad3-EPSM: T178V, S203A, S207A, S212A). Western blot analyses indicated that only Smad2/3-EPSM proteins accumulated onto chromatin, whereas wild-type Smad2/3 remained in the cytoplasm (Figure 6B). Similarly, Smad2/3-EPSM overexpressing hESCs were more prone to differentiate into endoderm when compared to late G1-hESCs overexpressing Smad2/3 (Figures 6C and 6D). Nevertheless, inhibition of CDK4/6 by PD0332991 improved endoderm differentiation of late G1-hESCs expressing Smad2 or Smad3 (Figure 6C). Finally, we analyzed the transcriptional activity of different forms of Smad2/3-EPSM mutants in late G1-hESCs taking advantage of the SEB4 reporter gene. These experiments revealed that phosphorylation of Smad2/3 at the linker region reduces their transcriptional activity (Figures 6E and 6F), whereas a reversion of specific phosphorylation sites to their wild-type residue (A212S, V178T) partially restored Smad3 sensitivity to cyclin D-CDK4/6-mediated phosphorylation (Figure 6E). These results demonstrate that cyclin D-CDK4/6 regulate Smad2/3 transcriptional activity in a cell-cycle phase-dependent manner by phosphorylating specific sites of the Smad2/3 linker regions to block their entry into the nucleus.

### Manipulating the Activity of Cyclin D-CDK4/6 Enables Differentiation of hIPSCs into Endoderm Cells without the Need of Exogenous Activin

**Figure 7:**
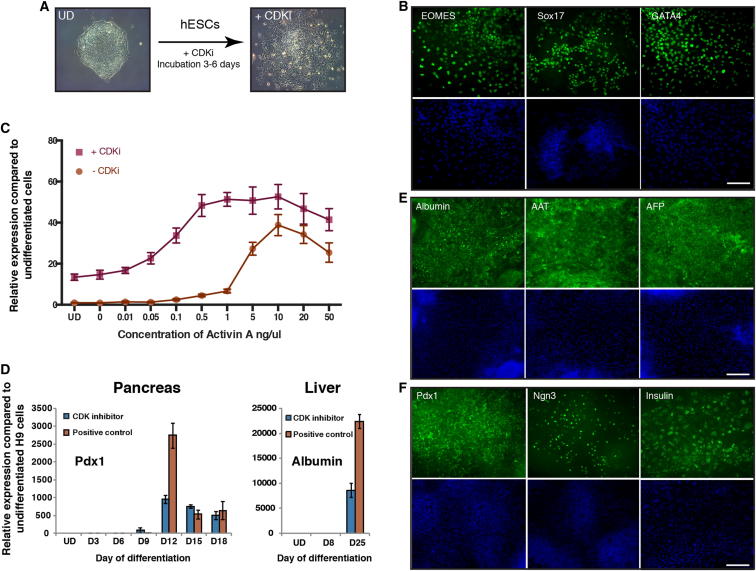
CDKi Treatment Induces Differentiation of hPSCs (A) Representative colonies of untreated hESCs and CDKi (0.75 μM PD0332991)-treated cells. (B) CDK4/6 inhibition results in endoderm differentiation. hESCs grown for 6 days in the presence of CDKi (0.75 μM PD0332991) were analyzed for the expression of germ layer markers using immunofluorescence microscopy. (C) CDK4/6 can replace Activin A during endoderm differentiation. H9 cells were incubated in the presence or absence of 0.75 μM CDKi in standard endoderm differentiation conditions and analyzed for Sox17 expression by qPCR. (D and E) Endoderm cells generated by CDKi can give rise to cells expressing hepatic markers. CDKi-produced endoderm was grown for 25 days in culture conditions for hepatic differentiation and then the expression of hepatocyte markers was analyzed using qPCR (D) or immunostaining (E). (F) Endoderm generated by CDKi can give rise to pancreatic cells. CDKi produced endoderm was grown for 18 days in culture conditions for pancreatic differentiation and then the expression of pancreatic markers was analyzed using qPCR (D) or immunostaining (F). Scale bar represents 100 μm. All data are shown as mean ± SD (n = 3). UD, undifferentiated cells. See also Figures S5, S6, S7, and Table S5.

**Figure S6:**
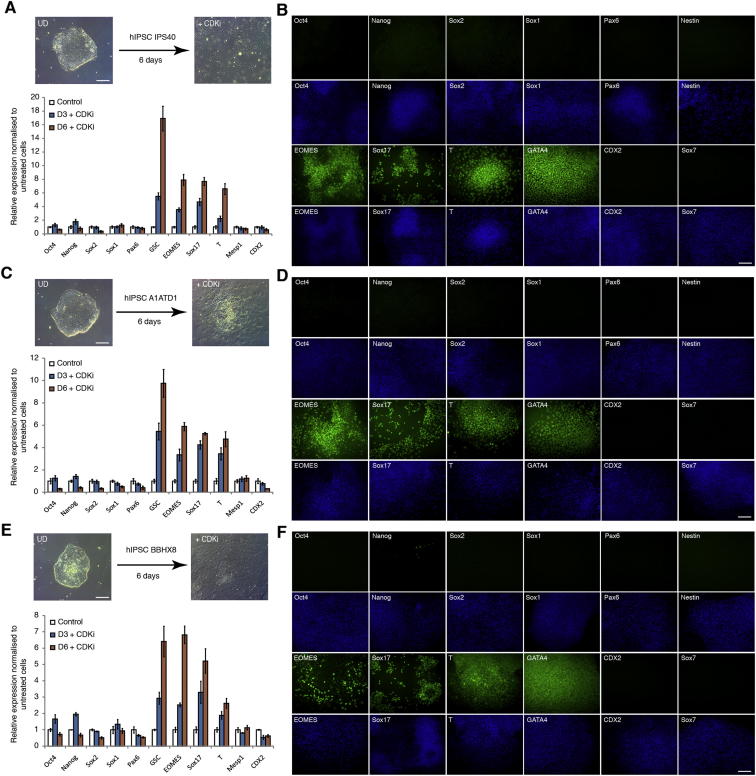
CDK4/6 Inhibition in hIPSCs Causes Endoderm Differentiation, Related to Figure 7 IPSCs were cultured in the presence of 0.75μM PD0332991 for 6 days and analyzed by (A) Q-PCR or (B) immunostaining in IPS40 cell line, by (C) Q-PCR or (D) immunostaining in A1ATD1 cell line and by (E) Q-PCR or (F) immunostaining in BBHX8 cell line. Data shown as mean ± SD (n = 3). Scale bar, 100μm.

**Figure S7:**
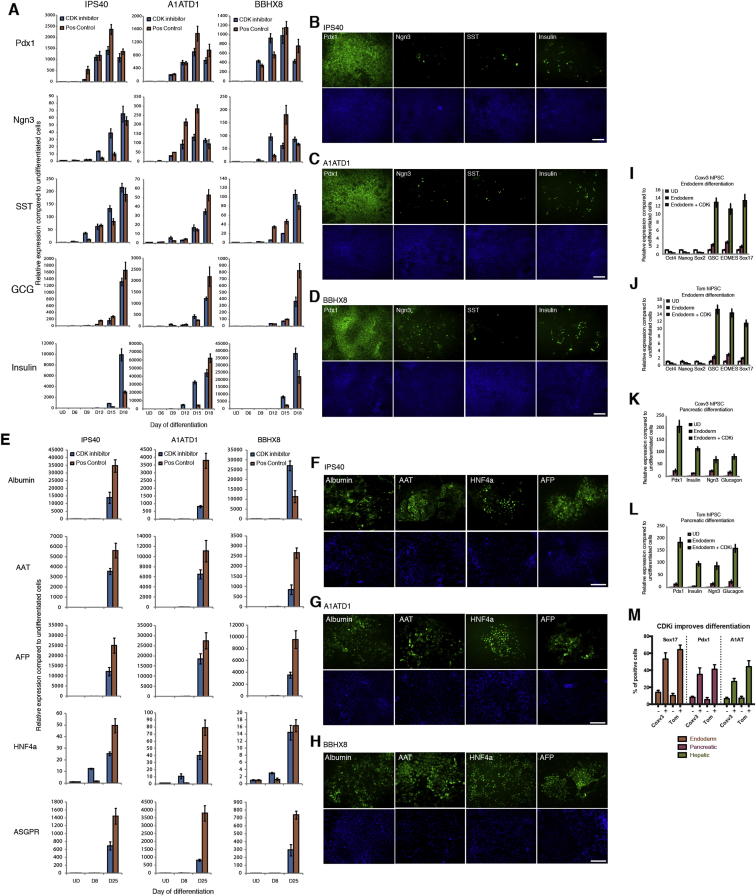
hIPSC CDKi-Produced Endoderm Can Give Rise to Pancreatic and Hepatic Cells, Related to Figure 7 (A–D) Analysis of pancreatic differentiation in hIPSCs. IPS40, A1ATD1 and BBHX8 cells were differentiated into endoderm with 0.75μM PD0332991 for 6 days and then further into pancreatic cells. Cells were analyzed by (A) Q-PCR or immunostaining at day 18 (day 12 for Pdx1) in (B) IPS40, (C) A1ATD1 or (D) BBHX8. Conventional endoderm differentiation protocol was used as a positive control. (E–H) Hepatic differentiation of endoderm cells generated from hIPSCs using CDKi. IPS40, A1ATD1 and BBHX8 cells were differentiated into endoderm with 0.75μM PD0332991 for 6 days and then further into hepatocytes. Cells were analyzed by (E) Q-PCR or by immunofluorescence microscopy at day 25 in (F) IPS40, (G) A1ATD1 or (H) BBHX8. Conventional hepatic differentiation protocol was used as a positive control. (I and J) CDK4/6 inhibition improves endoderm differentiation in endoderm-resistant hIPSCs Coxv3 and Tom. Cells were differentiated into endoderm by adding 0.75μM PD0332991 to conventional differentiation conditions for 6 days. (K and L) Cells were differentiated into endoderm with 0.75μM PD0332991 for 6 days and then further into pancreatic cells and analyzed by Q-PCR, or (M) by flow cytometry after endoderm, pancreatic or hepatic differentiation. Data shown as mean ± SD (n = 3). Scale bar, 100μm.

Interestingly, hESCs grown in the presence of PD0332991 gradually differentiated as shown by the decrease in pluripotency marker expression and by the increase in meso/endoderm markers (Figures 7A–7C and S5E; Table S5). In addition, we observed that endoderm cells induced by PD0332991 were able to differentiate further into hepatic and pancreatic progenitors (Figures 7D–7F and S5F). These observations prompted us to develop a protocol of endoderm differentiation taking advantage of the PD0332991 inhibitor. For that, we tested the effect of different combinations of Activin, BMP4, FGF2, and PD0332991 (Figure 7C and data not shown) on endoderm differentiation of hESCs and observed that this inhibitor decreased the need of exogenous Activin in our culture conditions. This study resulted in a protocol of endoderm differentiation in which exogenous Activin was replaced by PD0332991. To further validate this protocol, three different hIPSC lines were grown in defined culture conditions supplemented with PD0332991/FGF2/BMP4/LY29004 for 6 days. hIPSCs grown in these culture conditions differentiated into endoderm cells as shown by the decrease in pluripotency markers and the specific increase in endoderm markers (Figure S6). Furthermore, the resulting endoderm cells were able to differentiate further into cells expressing hepatic and pancreatic markers (Figures S7A–S7H). Finally, we also applied this protocol to hIPSCs resistant to conventional methods of differentiation and observed that chemical inhibition of CDK4/6-cyclin Ds significantly improved the capacity of these lines to differentiate into endoderm derivatives thereby allowing the generation of pancreatic and hepatic cells (Figures S7I–S7M). Considered together, these results show that manipulation of cyclin D-CDK4/6 activity can be used to direct differentiation of hPSCs toward the endoderm germ layers bypassing the need of exogenous Activin and the usual variability between lines. These results also showed that the function of cyclin D in cell fate choice of hESCs is conserved in hIPSCs and thus that the mechanisms revealed by our study could be applied to a broad number of human pluripotent stem cells.

## Discussion

Here, we have uncovered mechanisms by which the cell cycle can control the activity of signaling pathways directing cell fate choice. These mechanisms are orchestrated by cyclin D-CDK4/6 complex that limits the nuclear localization of key signaling effectors such as Smad2/3. This model of regulation implies that cell fate choice primarily occurs during the narrow window of G1 phase when cyclin D proteins are dynamically expressed. Accordingly, endoderm induction is only possible in early G1 when the level of cyclin D is low, allowing Smad2/3 to bind and to activate endoderm genes. Cyclin D expression in late G1 results in activation of CDK4/6 that bind Smad2/3 proteins and phosphorylate their linker region. The phosphorylation of Smad2/3 blocks their entry into the nucleus and thereby makes late G1 cells only receptive for neuroectoderm initiation. In agreement with the expression of cyclin D proteins, differentiation of ESCs is also accompanied by a change in G1 length (Coronado et al., 2013), and hESCs differentiating into endoderm display a longer G1 phase when compared to hESCs grown in culture conditions inductive for neuroectoderm specification (data not shown). Furthermore, lengthening of the G1 phase is associated with an increase in the population of hESCs in early G1 phase and thus with an increase in the number of cells specifically prone to endoderm differentiation. These observations imply that manipulating cell-cycle regulators could enable a controlled differentiation of stem cells. Accordingly, inhibiting CDK4/6 in hPSCs with a small molecule increases G1 length while inducing endoderm differentiation. Therefore, our study represents a first step toward the development of a method of differentiation in which simple manipulation of the cell cycle using small molecules could direct differentiation of pluripotent stem cells toward specific cell types without the need of exogenous growth factors.

Expression studies in the mouse embryo showed that cyclin Ds have a tissue-specific expression during gastrulation (Wianny et al., 1998). Mesoderm expresses cyclin D1/D2, neuroectoderm cyclin D1/D2, and endoderm a low level of cyclin D2, whereas cyclin D3 is specifically expressed in the trophectoderm. Therefore, the expression pattern of cyclin Ds during gastrulation mimic the expression of cyclin Ds during in vitro differentiation of hESCs, thereby suggesting that the mechanisms uncovered by our study might be transferable to the gastrulating embryo. However, genetic studies of individual cell-cycle regulators have been less conclusive and total absence of cyclin D in the mouse only leads to midgestation lethality (Kozar et al., 2004). Similar phenotypes were observed in embryos mutant for cyclin E or CDK2/4/6 suggesting that these factors are dispensable for early development. These results contrast with the broad number of studies that have shown the importance of these factors in established or primary cells in vitro. Furthermore, the importance of cell-cycle regulation in organogenesis has been identified in a diversity of systems (Fuchs, 2009, Li and Clevers, 2010). Therefore, it has been suggested that the absence of cyclin Ds in early mouse embryo could be rescued in part by CDK2-cyclin E that would compensate the absence of CDK4/6 activity by regulating the phosphorylation of pRB. Thus, the function of cyclin D in vivo could be masked by redundant and aberrant mechanisms. Species divergence between human and mice could also explain the apparent difference between our data generated with hESCs and the lack of cyclin D function in early mouse embryo. Indeed, there is growing evidence that part of the mechanisms controlling early development could differ between the two species. For instance, FGF signaling, which is known to promote proliferation, plays an essential role in specification of extraembryonic tissue in mouse blastocyst, whereas its inhibition has no effect in human embryo (Kuijk et al., 2012, Roode et al., 2012). Systematic studies on mEpiSCs could help to uncover the molecular basis for such differences and the existence of compensatory mechanisms specific to the mouse.

The interconnection of cell fate decisions and cell cycle revealed by our findings could also be relevant for stem cells in developing organs. Indeed, functional studies performed in the cortex and retina have demonstrated that loss of function of cyclin D/CDK results in the lengthening of G1 phase and is always accompanied by increased differentiation of neuronal stem cells specifically into neurons (Lange and Calegari, 2010, Lange et al., 2009). More recently, in vitro studies have shown that the length of G1 phase increases upon differentiation of neuronal stem cells in vitro, whereas inhibition of CDK4 induces their differentiation (Roccio et al., 2013). Similarly, absence of cyclin Ds or CDK4/6 results in premature differentiation of hematopoietic stem cells (Lange and Calegari, 2010). Considered together, these results suggest that the length of G1 phase, and thus cyclin D activity, defines the capacity of multipotent stem cells to differentiate in vivo. Such mechanisms would be essential to synchronize proliferation and differentiate tissue specific stem cells into particular lineages. Thus, our findings could not only start to uncover the molecular mechanisms that interconnect the cell cycle and cell fate decisions in hESCs but could also be relevant to understand how proliferation and differentiation of adult stem cells is coordinated in tissue homeostasis.

## Experimental Procedures

### Cell Culture of hESCs, hIPSCs, mEpiSCs, and mESCs

hESCs (H9 from WiCell) were used for all the experiments unless otherwise stated. H9 cells were grown in defined culture conditions as described previously (Brons et al., 2007). H9 cells were passaged weekly using collagenase IV and maintained in chemically defined medium (CDM) supplemented with Activin A (10 ng/ml) and FGF2 (12 ng/ml). hIPSCs (IPS40 and BBHX8; Vallier et al., 2009b; A1ATD1) were grown in culture conditions as described in the Extended Experimental Procedures.

### Differentiation of hESCs and IPSCs

hESCs were differentiated into neuroectoderm, endoderm, and mesoderm as described previously (Vallier et al., 2009b), hIPSCs and hESCs were differentiated into endoderm, pancreatic cells, and to hepatocytes as described in the Extended Experimental Procedures.

### Cell Sorting by FACS

FACS was performed as described before (Sakaue-Sawano et al., 2008). In sum, hESCs were washed with PBS and detached from the plate by incubating them for 10 min at 37°C in cell dissociation buffer (GIBCO). Cells were washed with cold PBS and then subjected to FACS with a Beckman Coulter MoFlo MLS high-speed cell sorter, using parameters described previously (Sakaue-Sawano et al., 2008).

Extended Experimental Procedures **Cell Culture of hESCs, hIPSCs, mEpiSCs, and mESCs**

hESCs (H9 from WiCell) and mEpiSCs were grown in defined culture conditions as described previously (Brons et al., 2007). H9 cells were passaged weekly and mEpiSCs every 5 days using collagenase IV and maintained in chemically defined medium (CDM) supplemented with Activin A (10 ng/ml) and FGF2 (12 ng/ml). hIPSCs (IPS40 and BBHX8 [Vallier et al., 2009a]; A1ATD1[Rashid et al., 2010]) were grown culture conditions as described before (Rashid et al., 2010).

**Differentiation of hESCs and IPSCs**

hESCs were differentiated into neuroectoderm, endoderm and mesoderm as described previously (Vallier et al., 2009b). Briefly, cells were cultured in CDM supplemented with SB-431542 (10 μM; Tocris) and FGF2 (12 ng/ml) for neuroectoderm, in CDM+PVA supplemented with Activin A (100 ng/ml), FGF2 (20 ng/ml), BMP4 (10 ng/ml), Ly294002 (10 μM; Promega) and CHIR99021 (3 μM; Selleck) for mesoderm and in CDM-PVA supplemented with Activin A (100 ng/ml), FGF2 (20 ng/ml), BMP4 (10 ng/ml) and Ly294002 (10 μM; Promega) for endoderm. hIPSCs were differentiated into endoderm and into hepatocytes as described before (Touboul et al., 2010). Pancreatic differentiation of hESCs and hIPSCs was carried out as follows. Daily media changes were made during the entire differentiation protocol. After endoderm differentiation, cells were cultured in Advanced DMEM (Invitrogen) supplemented with SB-431542 (10 μM; Tocris), FGF10 (50 ng/ml; AutogenBioclear), all-trans retinoic acid (RA, 2 μM; Sigma) and Noggin (50 ng/ml; R&D Systems) for 3 days. Cells were then cultured in Advanced DMEM + human FGF10 (50 ng/ml; AutogenBioclear), all-trans retinoic acid (RA, 2 μM; Sigma), KAAD-cyclopamine (0.25 μM; Toronto Research Chemicals) and Noggin (50 ng/ml; R&D Systems) for 3 days. Next, cells were cultured in human KGF (50 ng/ml; R&D Systems) for 3 days. For maturation of pancreatic progenitors, cells were grown in Advanced DMEM + 1% vol/vol B27 and DAPT (1 mM) for 3 days and for 3 additional days in Advanced DMEM + 1% vol/vol B27.

**qPCR and Immunostaining**

Methods for Q-PCR and immunostaining have been described previously (Vallier et al., 2009b). Q-PCR data are presented as the mean of three independent experiments and error bars indicate standard deviations. Primer sequences and antibodies have been listed in Extended Experimental Procedures.

**Generating Fucci-hESCs**

Human mKO2-Cdt1 and mAG-Geminin fusion sequences were inserted into the pTP6 plasmid, so that mKO2-Cdt1 construct contained a selection marker for G418 and mAG-Geminin for puromycin. Constructs were verified by sequencing. Stable H9 Fucci-hESC lines were then generated with mKO2-Cdt1, mAG-Geminin or with both mKO2-Cdt1/mAG-Geminin as follows. Constructs were transfected (a simultaneous transfection with both constructs for double mKO2-Cdt1/mAG-Geminin cell line) into H9 hESCs with lipofectamine as described previously (Vallier et al., 2004) and grown for 3 days. Cells were then cultured in the presence of appropriate antibiotics (0.2 mg/ml G418 for and 1 mM for puromycin) until the emergence of resistant colonies. Clones were individually picked and further characterized for the expression of the Fucci reporter proteins (Figure 1).

**Generating Cyclin D Single-Knockdown, Double-Knockdown, and Triple-Knockdown Cells**

For cyclin D single knockdown, previously validated shRNA expression vectors (Open Biosystems, Cat no. RHS4533-NM053056, RHS4533-NM001759, RHS4533-NM001136017) directed against cyclin D1, D2 or D3 were transfected into H9 hESCs with lipofectamine (Vallier et al., 2004) and grown for 3 days (Figure S3). Cells were then cultured in the presence of puromycin until antibiotic resistant colonies appeared. These were picked and characterized for knockdown efficiency (Figure S3). For cyclin D double knockdown, single knockdown sublines were stably transfected with a second shRNA expression vector directed against a different cyclin D and containing a hygromycin resistance gene. Double knockdown cells were cultured in the presence of puromycin and hygromycin until colonies appeared. These were picked and characterized for knockdown efficiency (Figure S3). For cyclin D triple knockdown, cyclin D double knockdown cells were transitorily transfected with an shRNA expression vector directed against the third cyclin D family member and containing eGFP as a marker for transfected (cyclin D triple knockdown) cells. eGFP cell were either sorted for Q-PCR, western blot or analyzed directly by immunostaining.

**Generating Cyclin D Overexpressing Cells and Cyclin D1 Mutant Cells**

cDNA of cyclin D1, D2, D3, D1 T156A and D1 T286A was cloned into the pTP6 vector (Pratt et al., 2000) with an N-terminal FLAG-HA tag, under the regulation of CAG promoter. The inserts were confirmed by sequencing. Vectors were transfected into H9 hESCs by lipofection (Vallier et al., 2004) and grown for 3 days (Figure S4). Thereafter, cells with a stable integration were selected by continuous presence of puromycin. Individual clones were picked, propagated and analyzed for subsequent analyses.

**Cell Sorting by FACS**

FACS was performed as described before (Sakaue-Sawano et al., 2008). In sum, hESCs were washed with PBS and detached from the plate by incubating them for 10 min at 37°C in Cell Dissociation Buffer (GIBCO). Cells were washed with cold PBS and then subjected to FACS with a BeckmanCoulter MoFlo MLS high-speed cell sorter, using parameters described previously (Sakaue-Sawano et al., 2008).

**Luciferase Assay**

Cells were transfected with a Smad2/3 reporter construct (SBE4-luciferase), Sox17 or GSC promoter constructs (Brown et al., 2011) and Renilla luciferase at a ratio of 10:1, using Lipofectamine 2000 (Invitrogen) (Vallier et al., 2004). Luciferase activity was measured with the dual luciferase assay kit following (Promega) manufacturer instructions. Firefly luciferase activity was normalized to Renilla luciferase activity for cell numbers and transfection efficiency. Samples were analyzed on a Glomax Luminometer and software.

**Time-Lapse Imaging**

Cells were grown in Chambered 1.0 Borosilicate Cover Glass System (Lab-TEK). Time-lapse imaging of cells was carried out with Leica SP5 invert + live cell chamber x2 confocal microscope, using parameters as described previously (Sakaue-Sawano et al., 2008). Cells were maintained in the presence of CO2 at 37°C during microscopy.

**Chromatin Immunoprecipitation**

hESCs were washed with PBS and detached from the plate by incubating them for 10 min at 37°C in Cell Dissociation Buffer (GIBCO). ChIP was carried out as described before (Brown et al., 2011), except that crosslinking was performed in solution in PBS if samples were sorted by FACS.

**Cell Fractionations**

Cells were harvested with trypsin and washed twice with cold PBS. For cytoplasmic lysis, cells were suspended in 5 times packed cell volume (1 μl PCV = 10^6^ cells) equivalent of Isotonic Lysis Buffer (10 mM Tris HCl, pH 7.5, 3 mM CaCl, 2 mM MgCl_2_, 0.32 M Sucrose, Complete protease inhibitors and phosphatase inhibitors), and incubated for 12 min on ice. Triton X-100 was added to a final concentration of 0.3% and incubated for 3 min. The suspension was centrifuged for 5 min at 1,500 rpm at 4°C and the supernatant (cytoplasmic fraction) transferred to a fresh chilled tube. For nuclear lysis, nuclear pellets were resuspended in 2 x PCV Nuclear Lysis Buffer+Triton X-100 (50 mM Tris HCl, pH 7.5, 100 mM NaCl, 50 mM KCl, 2 mM MgCl_2,_ 1 mM EDTA, 10% Glycerol, 0.3% Triton X-100, Complete protease inhibitors and phosphatase inhibitors) and dounce homogenized. The samples were incubated with gentle agitation for 30 min at 4°C and then centrifuged with a Ti 70.1 rotor at 22,000 rpm for 30 min at 4°C or with a Ti 45 rotor for 30 min at 20,000 rpm at 4°C. The chromatin pellets were dounce homogenized in 2 x PCV Nuclear Lysis Buffer+Triton X-100 and Benzonase until the pellets gave much less resistance. The samples were incubated at RT for 30 min and centrifuged with either a Ti 70.1 rotor for 30 min at 22,000 rpm at 4°C or with a Ti 45 rotor for 30 min at 20,000 rpm at 4°C.

**Protein Coimmunoprecipitation**

Antibodies were crosslinked to Protein G-Agarose beads (Roche, 1 μg of antibody per 5 ul of beads) with dimethyl pimelimidate (Sigma) using standard biochemical techniques, prior to performing immunoprecipitations. Samples were incubated with 5 μg of crosslinked antibodies for 12h at 4°C. Beads were washed five times with ten bead volumes of Nuclear Lysis Buffer and eluted in SDS western blotting buffer (30 mM Tris pH 6.8, 10% Glycerol, 2% SDS, 0.36 M beta-mercaptoethanol (Sigma), 0.02% bromophenol blue) by heating at 90°C for 5 min. Samples were analyzed by standard western blotting techniques.

**Flow Cytometry**

Flow cytometry was carried out with a BD MoFlo flow cytometer and analyzed by FloJo software. Cell-cycle distribution was analyzed by Click-It EdU incorporation Kit (Invitrogen) according to manufacturer’s guidelines. Marker expression was analyzed at various time points during differentiation by first dissociating cells into single cells with Cell Dissociation Buffer (GIBCO) and fixing in 4% PFA for 20 min at 4°C. This was followed by permeabilization and blocking with 10% serum + 0.1% Triton X-100 in PBS for 30 min at RT and incubation with primary antibody in 1% serum + 0.1% Triton X-100 for 2h at 4°C. After washing the samples three times with PBS, they were incubated with a secondary antibody for 2h at 4°C, washed three times with PBS and analyzed by flow cytometry.

## Supplemental Information

Document S1. Tables S1–S5

Document S2. Article plus Supplemental Information
