# Supplementary material for: The Cell-Cycle State of Stem Cells Determines Cell Fate Propensity
Source: Cell. 2013 Sep 26;155(1):135–47. doi: 10.1016/j.cell.2013.08.031 (PMC3898746; doi:10.1016/j.cell.2013.08.031)
Supplement: Document S1. Tables S1–S5 [file mmc1.pdf]

Table S1. Q-CPR Primers, Related to Experimental Procedures

Gene	Primer name	Sequence
PBGD	PBGDF	GGAGCCATGTCTGGTAACGG
	PBGDR	CCACGCGAATCACTCTCATCT
Oct4	Pou5F_F	AGTGAGAGGCAACCTGGAGA
	Pou5F_R	ACACTCGGACCACATCCTTC
Nanog	NanogF	CATGAGTGTGGATCCAGCTTG
	NanogR	CCTGAATAAGCAGATCCATGG
Sox2	hSox2F	TGGACAGTTACGCGCACAT
	hSox2R	CGAGTAGGACATGCTGTAGGT
Nestin	NestinF	GAAACAGCCATAGAGGGCAAA
	NestinR	TGGTTTTCCAGAGTCTTCAGTGA
Olig3	Olig3F	AGCCGTCTCAACTCGGTCT
	Olig3R	CATGGCTAGGTTCAGGTCGTG
GBX2	GBX2F	GTTCCACTGCAAAAAGTACCTCT
	GBX2R	GGGACGACGATCTTAGGGTTC
Sip1	Sip1F	CGCTTGACATCACTGAAGGA
	Sip1R	CTTGCCACACTCTGTGCATT
Pax6	hPax6F	CTTTGCTTGGGAAATCCGAG
	hPax6R	AGCCAGGTTGCGAAGAACTC
Sox1	Sox1Q	QUANTITECT PRIMERS (QIAGEN)
GSC	DAGSCF	GAGGAGAAAGTGGAGGTCTGGTT
	DAGSCR	CTCTGATGAGGACCGCTTCTG
Brachury	DABraF	TGCTTCCCTGAGACCCAGTT
	DABraR	GATCACTTCTTTCCTTTGCATCAAG
Eomes	hEomesF	ATCATTACGAAACAGGGCAGGC
	hEomesR	CGGGGTTGGTATTTGTGTAAGG
Sox17	hsox17F	CGCACGGAATTTGAACAGTA
	hsox17R	GGATCAGGGACCTGTCACAC
MixL1	hMixL1F	GGTACCCCGACATCCACTTG
	hMixL1R	TAATCTCCGGCCTAGCCAAA
Mesp1	Mesp1F	GAAGTGGTTCCTTGGCAGAC
	Mesp1R	TCCTGCTTGCCTCAAAGTGT
Mesp2	Mesp2F	AGCTTGGGTGCCTCCTTATT
	Mesp2R	TGCTTCCCTGAAAGACATCA
Flk-1	Flk1F	TTTTTGCCCTTGTTCTGTCC
	FLK1R	TCATTGTTCCCAGCATTTCA
Tbx6	Tbx6F	AAGTACCAACCCCGCATACA
	Tbx6R	TAGGCTGTCACGGAGATGAA
Hoxb1	Hoxb1F	AGAACCGACGAATGAAGCAG

	Hoxb1R	GACTGGTCTGAGGCATCTCC
Hand1	HAND1 F	GTGCGTCCTTTAATCCTCTTC
	HAND1 R	GTGAGAGCAAGCGGAAAAG
CCND1	CCND1F	GCCGTCCATGCGGAAGATC
	CCND1R	CCTCCTCCTCGCACTTCTGT
CCND2	CCND2F	TGAGGCGGTGTAGGACAGG
	CCND2R	ATATCCCGCACGTCTGTAGG
CCND3	CCND3F	CATGTACCCGCCATCCAT
	CCND3R	AGCTTCGATCTGCTCCTGAC

Table S2. CHIP Primers, Related to Experimental Procedures

Gene	Primer name	Protein CHIP	Sequence
EOMES	C_eomes1aF	Smad2/3 CHIP	GTTCCGGCCGATTACTTGTC
	C_eomes1aR	Smad2/3 CHIP	GGGAGGCTTCCGACTACTCA
Mixl1	C_MixL11F	Smad2/3 CHIP	TTTGATGAGGACAGACGGGA
	C_MixL11R	Smad2/3 CHIP	CGAAGGACTATTTGCCTGGG
GSC	C_GSC1F	Smad2/3 CHIP	GTGCAGGGCACAGTTCAGAG
	C_GSC1R	Smad2/3 CHIP	TATGGGACGCTTTGAATCCC
Neg Control	Smad7-veF	Smad2/3 and Cyclin D CHIP	ACCCTGATAGGAAGAGGGGAAG
	Smad7-veR	Smad2/3 and Cyclin D CHIP	TCACACACACTCCTGACAAGTGA

Table S3. Antibodies, Related to Experimental Procedures

Antibody raised against	Catalog number	Company
Actin, clone C4	MAB1501	Chemicon
Brachyury (T)	af2085	R&D Systems
Cdx2	MU392A-UC	BioGenex
Cyclin D1 (DCS-6)	sc-20044	Santa Cruz
Cyclin D1	sc-246	Santa Cruz
Cyclin D2 (C-17)	sc-181	Santa Cruz
Cyclin D3 (D-7)	sc-6283	Santa Cruz
Cyclin D3	sc-6283	Santa Cruz
EOMES	ab23345	R&D Systems
Gata4 (G-4)	sc-25310	Santa Cruz
Histone H3	ab1791	Abcam
Nanog	af1997	R&D Systems
Nestin (Rat-401)	sc-33677	Santa Cruz
Oct-3/4 (C-10)	sc-5279	Santa Cruz
Pax6	PRB-278P-100	Covance
P300	sc-584	Santa Cruz
Sox1	AF3369	R&D Systems
Sox17	AF1924	R&D Systems
Sox2	AF2018	R&D Systems
Sox7	AF2766	R&D Systems
CXCR4	MAB173	R&D Systems
Tra-1-60	sc-21705	Santa Cruz
Smad2/3	sc-8332	Santa Cruz
Smad2/3	AF3797	R&D Systems
Smad3 (phospho-Thr179)	A1003-ABT	Assay Biotech
Smad3 (phospho-Thr204)	A1004-ABT	Assay Biotech
Smad3 (phospho-Thr208)	A1005-ABT	Assay Biotech
Smad3 (phospho-Thr213)	A1007-ABT	Assay Biotech
Smad2 (phospho-Thr220)	A1000-ABT	Assay Biotech
Smad2 (phospho-Ser250)	A0998-ABT	Assay Biotech
Alexa Fluor 647 donkey α-mouse	A31571	Invitrogen
Alexa Fluor 647 donkey α-goat	A21447	Invitrogen

Table S4. Overall Cell Output in Cyclin D Double Knockdown or Cyclin D Overexpressing Cells after Endoderm, Mesoderm, and Neuroectoderm Differentiation, Related to Figure S3

		Total cell number per cm2	+/-	Total cell number per cm2	+/-	Total cell number per cm2	+/-	Total cell number per cm2	+/-
		Scramble		D1D3		D1D2		D2D3	
Cyclin D Double KD	Undiffe-rentiated	119658	6352	108568	5260	115279	7128	122684	7844
	Endoderm	84711	5367	72636	5399	70336	6704	65614	3250
	Mesoderm	367895	31389	298670	17241	252014	14193	222073	15957
	Neuro-ectoderm	137179	9127	96574	9102	53783	3898	40466	2290
		GFP		Cyclin D1		Cyclin D2		Cyclin D3	
Cyclin D OE	Undiffe-rentiated	124497	7530	110680	6846	102970	8254	94378	5612
	Endoderm	90763	6936	58190	3679	39878	1695	30311	2227
	Mesoderm	131703	6243	88953	6324	58702	4943	41949	3868
	Neuro-ectoderm	269605	16831	278489	11727	278340	15982	247273	15389

Table S5. CDK4/6 Inhibition Does Not Significantly Alter the Cell Output during Differentiation, Related to Figure 7

		- CDKi		+ CDKi	
		Total cell number per cm2	+/-	Total cell number per cm2	+/-
H9	Undifferentiated	113526	5624	113526	5624
	Endoderm	82632	6141	72173	4500
	Pancreatic	302632	25491	258546	26973
	Hepatic	140789	15818	115627	8362
Bob7	Undifferentiated	95789	6396	95789	6396
	Endoderm	76579	5225	65416	6825
	Pancreatic	253947	23930	234229	19289
	Hepatic	134263	18441	105063	7505
BBHX8	Undifferentiated	108026	4433	108026	4433
	Endoderm	85526	6784	87941	7672
	Pancreatic	284211	21129	219825	12874
	Hepatic	137368	14609	116112	9575
IPS40	Undifferentiated	98316	5106	98316	5106
	Endoderm	72632	6990	66324	5058
	Pancreatic	268421	16032	226306	11932
	Hepatic	125263	9185	109284	13394

Cells were differentiated into endoderm with CDK4/6 inhibitor and further to pancreatic and hepatic cells in H9, Bob7, BBHX8 or IPS40 cells and then counted to estimate cell numbers.
